# Impact of proton therapy on the DNA damage induction and repair in hematopoietic stem and progenitor cells

**DOI:** 10.1038/s41598-023-42362-0

**Published:** 2023-10-09

**Authors:** Simon Sioen, Oniecha Vanhove, Barbara Vanderstraeten, Carlos De Wagter, Monique Engelbrecht, Charlot Vandevoorde, Evan De Kock, Marc-Jan Van Goethem, Anne Vral, Ans Baeyens

**Affiliations:** 1 Radiobiology, Department of Human Structure and Repair, Faculty of Medicine and Health Sciences, Ghent University, Corneel Heymanslaan 10, 9000 Ghent, Belgium; 2 Medical Physics, Department of Human Structure and Repair, Faculty of Medicine and Health Sciences, Ghent, Belgium; 3 Department of Radiotherapy-Oncology, Ghent University Hospital, Ghent, Belgium; 4 Separated Sector Cyclotron Laboratory, Radiation Biophysics Division, iThemba LABS (NRF), Cape Town, 7131 South Africa; 5 Biophysics Department, GSI Helmholtzzentrum für Schwerionenforschung, Darmstadt, Germany; 6 Department of Radiation Oncology and Particle Therapy Research Center, University Medical Center Groningen, University of Groningen, Groningen, The Netherlands; 7 Cancer Research Institute Ghent (CRIG), Ghent, Belgium

**Keywords:** Chromosomes, DNA damage and repair, Cancer therapy, Cancer, Cell biology, Molecular biology

## Abstract

Proton therapy is of great interest to pediatric cancer patients because of its optimal depth dose distribution. In view of healthy tissue damage and the increased risk of secondary cancers, we investigated DNA damage induction and repair of radiosensitive hematopoietic stem and progenitor cells (HSPCs) exposed to therapeutic proton and photon irradiation due to their role in radiation-induced leukemia. Human CD34^+^ HSPCs were exposed to 6 MV X-rays, mid- and distal spread-out Bragg peak (SOBP) protons at doses ranging from 0.5 to 2 Gy. Persistent chromosomal damage was assessed with the micronucleus assay, while DNA damage induction and repair were analyzed with the γ-H2AX foci assay. No differences were found in induction and disappearance of γ-H2AX foci between 6 MV X-rays, mid- and distal SOBP protons at 1 Gy. A significantly higher number of micronuclei was found for distal SOBP protons compared to 6 MV X-rays and mid- SOBP protons at 0.5 and 1 Gy, while no significant differences in micronuclei were found at 2 Gy. In HSPCs, mid-SOBP protons are as damaging as conventional X-rays. Distal SOBP protons showed a higher number of micronuclei in HSPCs depending on the radiation dose, indicating possible changes of the in vivo biological response.

## Introduction

Radiotherapy plays an important role, next to chemotherapy and surgery, in treating childhood cancers^1–3^*.* Normal tissue damage and secondary cancer risk from radiotherapy of primary cancers in adults are small compared to the benefits from radiotherapy. Unfortunately, children are at a greater risk than adults for developing cancer after being exposed to ionizing radiation, which is mainly due to the higher rate of tissue development and regeneration in children^4,5^. When individuals are exposed during childhood, the risk to develop radiation-induced soft tissue sarcoma, thyroid cancer, breast cancer or leukemia is significantly higher compared to adults^4,5^.

The high prevalence of radiation-induced leukemia in children is related to the high radiosensitivity of the hematopoietic tissue at young ages, particularly the bone marrow which harbors hematopoietic stem and progenitor cells (HSPCs)^6^. HSPCs lie at the top of the hierarchic hematopoietic system where they have clonogenic potential and are capable of self-renewal and differentiation into all mature blood cells. HSPCs may be exposed in vivo to radiation during therapy either directly, when part of the bone marrow is irradiated, or indirectly, when HSPCs present in peripheral blood pass the radiation field. The specific characteristics of HSPCs make them a major target for radiation-induced normal tissue toxicity and possible malignant transformation as radiation-induced DNA damage may propagate genomic damage across the whole hematopoietic system^7,8^. In addition, the quiescent nature of HSPCs leads to an elevated vulnerability to mutagenesis due to the preference of error-prone non-homologous end-joining to repair double-stranded breaks (DSB)^7,9^.

In children, central nervous system (CNS) tumors represent 15–20% of all malignant neoplasms and are the most frequent solid tumors in the pediatric age as well as the leading cause of cancer-related morbidity and mortality^10^. In these patients, approximately 20–30% of the active bone marrow is located in the cranium and cervical vertebrae^11^. Therefore, it is important to take red bone marrow into account during treatment of CNS tumors, as craniospinal irradiation is often standard of care in these patients^12^. This is further emphasized by the fact that bone marrow toxicity from radiation may limit subsequent local or systemic treatment regimens^13^. As the focus of most research today is to maintain or improve survival rates while attempting to reduce or eliminate long-term morbidities for each specific patient, radiotherapeutic treatments such as proton therapy are currently on the rise, in particular for treatment of childhood brain tumors^11,14,15^. Protons are ionizing charged particles and are different from high-energy X-rays, which are commonly used in radiotherapy^11^. While penetrating tissues, protons are slowed down and deposit most of their energy at the end of their range, which leads to a well-defined characteristic depth-dose distribution, called the Bragg peak. By modifying the energy of accelerated protons, the range of penetration can be adjusted. This allows for combination of multiple Bragg peaks into a spread-out-Bragg peak (SOBP), that encompasses the whole tumor, while reducing normal tissue doses^16^. This differs from conventional X-ray radiotherapy, where a relatively high entrance and exit dose are still present which may lead to toxicity problems for the healthy tissue surrounding the tumor and limits the ability to increase the tumor dose^16,17^.

Although proton therapy is now widely used to treat numerous cancers patients at over 98 clinically operational facilities, there are still hurdles to overcome in terms of physical dose delivery as well as unknowns about the radiobiology of protons^16,18^. The key metric by which proton and photon radiotherapy differ is the linear energy transfer (LET). LET is defined as the amount of energy per unit distance that is transferred to the surrounding medium by a particle along its trajectory (keV/µm) and is increasing along the depth of the SOBP^19^. The region of maximum LET is therefore located at the distal part of the SOBP^20,21^. A higher LET leads to a higher ionization density, leading to more complex DNA damage which is therefore more difficult to repair resulting in a higher yield of chromosomal aberrations. This has been seen in various in vitro studies^22–25^. To describe the biological effect of proton radiation, the proton relative biological effectiveness (RBE) is used. The proton RBE is the ratio of the absorbed doses that produce the same biological effect between a reference radiation (6 MV X-rays) and proton radiation^26^. In the clinic, a fixed RBE value of 1.1 for protons is typically used, which is based on experimental data and the fact that LET variations are not modeled in clinical treatment planning^26–28^. While there is evidence for a variable RBE along the proton beam depth, this remains a topic of active debate^20,29^. In particular, near the distal region of the Bragg peak several in vitro and in vivo studies showed a significantly higher RBE due to the higher LET, which can be critical as this SOBP portion is likely to be located in healthy tissue^23,25,30–32^. These recent findings question the accuracy of a fixed RBE value along the proton beam with respect to treatment safety and efficacy.

As exposure to radiation, particularly for pediatric cancer patients, is clearly correlated to a higher risk of leukemogenesis and as the proton biological effectiveness is still being questioned, it is crucial to investigate the radiation response of HSPCs in the light of the rapidly growing application of proton therapy. Multiple studies have suggested differences in DNA repair, cell survival and cell cycle perturbations between proton or photon therapy in different cells^33,34^. More specifically for human HSPCs, several studies on DNA damage response have been published with other radiation types but not with protons^35–37^. In this study, we investigated the differences in DNA damage induction and repair of human CD34^+^ cells, isolated out of umbilical cord blood (UCB), after exposure to high energy photon and proton irradiation at different SOBP positions by means of the γ-H2AX foci assay and the cytogenetic cytokinesis-block micronucleus assay (Supplementary file 1).

## Results

**Figure 1:**
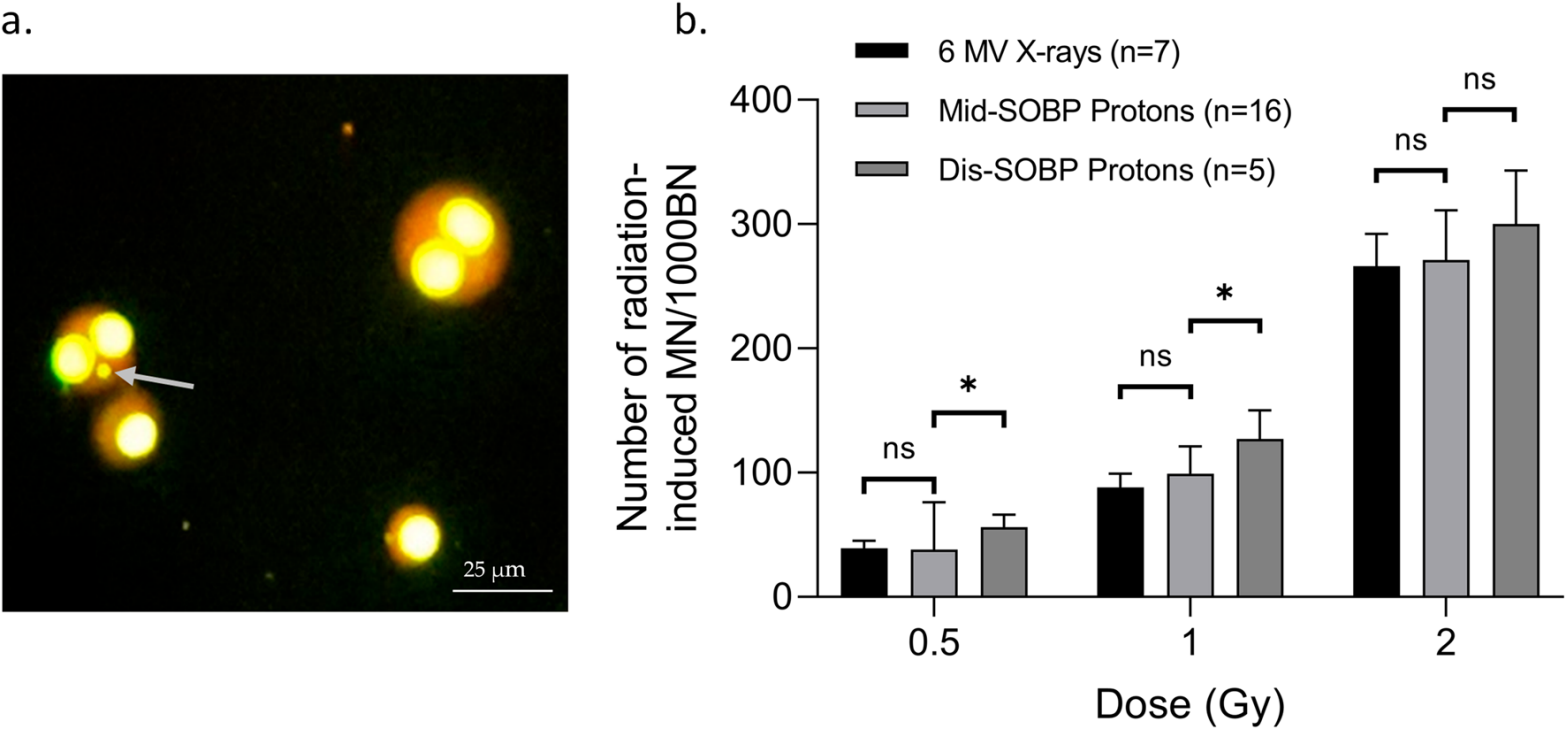
(**a**) Image of mono and bi-nucleated (BN) CD34^+^ HPSCs at 400 × magnification. The grey arrow indicates a micronucleus. (**b**) The graph represents the number of radiation-induced micronuclei (MN) per dose (Gy) scored in 1000 binucleated (BN) CD34^+^ HSPCs, irradiated with 6 MV X-rays, mid- spread-out Bragg peak (SOBP) protons or distal-SOBP protons, 70 h post-irradiation. The number of donors per condition is shown. Error bars represent the standard deviation of the mean of the donors. Significance is indicated (*p < 0.05).

The MN, representing chromosomal damage, were counted. As shown in Fig. 1, the number of MN counted in 1000 BN cells showed no significant differences between 6 MV X-rays and mid-SOBP protons at each dose. CD34^+^ HSPCs were also exposed to distal SOBP protons at the PARTREC facility. When comparing their respective MN data to mid-SOBP protons and 6 MV X-rays, a higher number of MN/1000BN cells was found for the distal SOBP-exposed CD34^+^ HSPCs. This difference was significant for the 0.5 and 1 Gy exposure but not for the 2 Gy exposure. Hence, the proton biological enhancement ratios for distal SOBP protons are higher (up to 1.4) and the proton biological enhancement ratios for mid-SOBP are as expected (1.0–1.1) (Table 1).

**Table 1:** Calculated biological enhancement ratio’s (BER) values for mid- and distal spread-out Bragg peak (SOBP) proton irradiations, relative to the reference 6 MV X-ray irradiations.

	Dose (Gy)	BER mid-SOBP	BER distal SOBP
Micronucleus assay	0.5	1.0	1.4
	1.0	1.1	1.4
	2.0	1.0	1.1
γ-H2AX foci assay (30 min)	1.0	1.0	1.0

γ-H2AX foci were visualized by immunofluorescence, to detect the number of radiation-induced DSBs, whereby each focus theoretically represents one DSB. The analysis of foci, at different time points post-irradiation, gives an estimation of the induction and repair of DNA DSBs, whereby the amount of foci linearly increases with increasing dose and declines as a function of time post-irradiation^38^.

**Figure 2:**
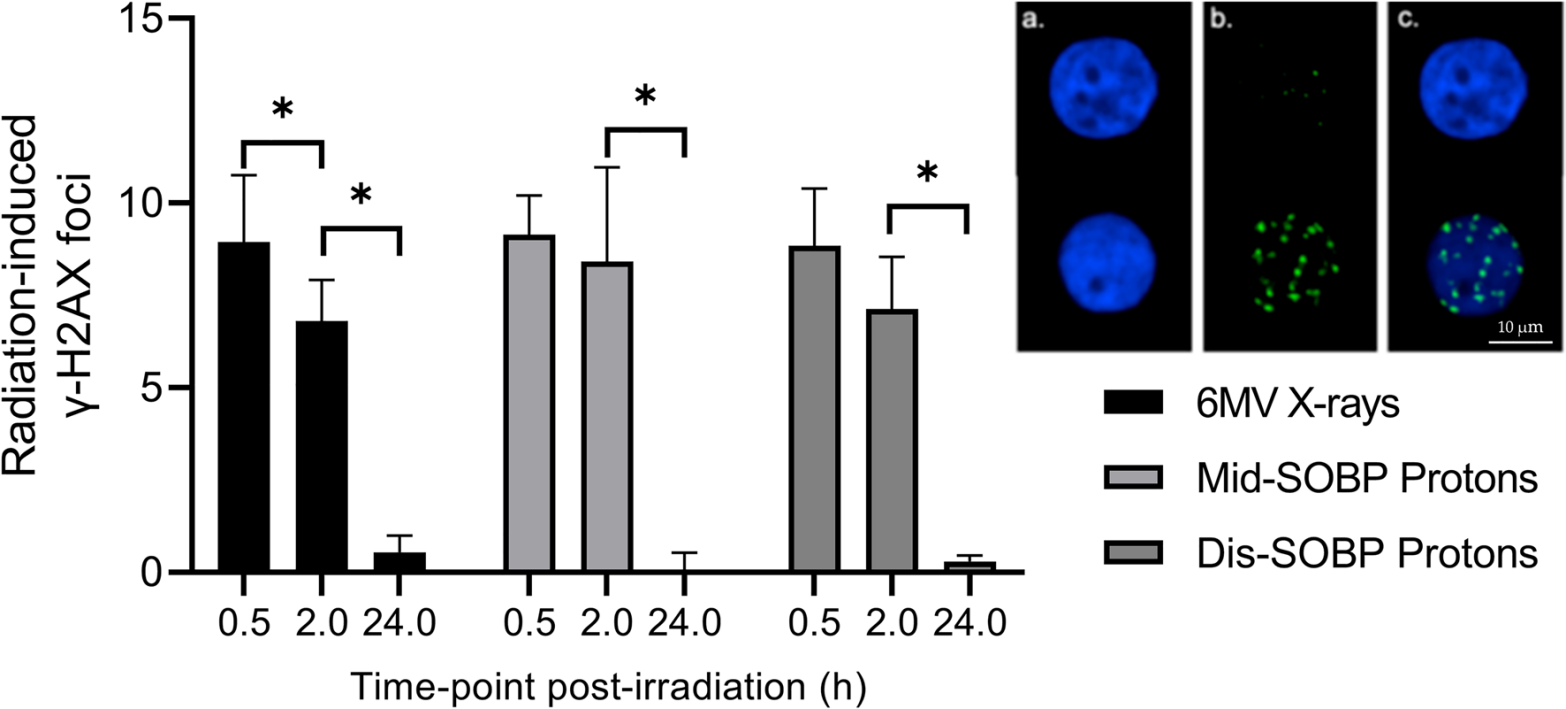
Image of the γ-H2AX foci present on a sham-irradiated (top) and an irradiated (bottom) CD34^+^ HSPCs: (**a**) 4',6-diamidino-2-fenylindool (DAPI) staining (**b**) γ-H2AX foci on the fluorescein isothiocyanate (FITC) channel **c.** Merged picture. Graph shows the mean radiation-induced γ-H2AX foci in CD34^+^ HSPCs at 30 min, 2 h and 24 h post-irradiation exposure to 6 MV X-rays, mid- spread-out Bragg peak (SOBP) and distal SOBP protons. Error bars represent the standard deviation of the mean over the donors (n = 5). Significance is indicated (*p < 0.05).

As represented in Fig. 2, the number of initial radiation-induced γ-H2AX foci at 30 min and residual radiation-induced γ-H2AX foci at 2 h post-exposure showed no significant differences between 6 MV X-ray, mid- or distal-SOBP protons. Also, residual radiation-induced γ-H2AX foci at 24 h post-exposure, indicating residual DNA DSBs, showed no significant differences between 6 MV X-ray, mid- or distal-SOBP protons (Fig. 2). This was also represented by a proton biological enhancement ratio of 1 for both mid- and distal SOBP protons, relative to the 6 MV X-ray exposures (Table 1). For each condition, significant differences were found between the radiation-induced γ-H2AX foci at 2 h and 24 h. Only for the 6 MV condition, a significant difference was found between 30 min and 2 h.

## Discussion

In this study, we showed that the same number of initial double-stranded breaks (DSBs) were induced by exposure to 6 MV X-rays, mid- or distal SOBP protons, based on the number of γ-H2AX foci at 30 min. Also, at 2 h and 24 h post-irradiation no statistical differences were found between these conditions. Additionally, when comparing the number of radiation-induced DSBs between the different time points, all condition showed a significant difference between 30 min and 24 h, indicating DSB repair. Only the 6 MV X-ray condition showed a significant difference between 30 min and 2 h per condition. The significantly higher number of radiation-induced MN for distal SOBP protons compared to mid-SOBP protons and the higher biological enhancement ratio reflected a higher number of residual DNA damage at the distal end of the proton beam. The fact that no change in the number of γ-H2AX foci was observed at 30 min and 24 h, while a higher number of MN was observed after exposure to distal-SOBP compared to mid-SOBP protons, might be explained by a higher complexity of the DSBs induced by distal-SOBP protons. As mentioned before, this outcome has been seen in various in vitro studies and is linked to the increased LET in this region^22–25^.

However, RBE and their analogues do not only depend on LET but also on different biological properties such as the studied endpoint, the tissue type or cell line and the cell cycle stage^39^. Furthermore, RBE is also influenced by multiple physical properties such as the proton beam energy, dose and dose rate^40^. The obtained proton biological enhancement ratios can be used to further optimize RBE models, where RBE-weighted doses (D_RBE_) are used to incorporate differential RBEs in the irradiated volume. These RBE models are implemented in treatment plan optimization and evaluation as the use of generic RBEs may lead to inaccurate toxicity estimations^41^. Ultimately, for an optimal clinical use of proton therapy, approaches to limit treatment toxicity should be further developed while biological advantages of protons should be exploited^42^.

To further investigate the DNA damage response in HSPCs after irradiation, cell death analysis could be performed. Normal tissue injury during and following radiotherapy has been attributed to the loss of regenerative capacity, cell death and senescence in the hematopoietic stem cell compartment^8^. Besides apoptosis, novel highly controlled cell death pathways are emerging, which can be induced by radiotherapy^43,44^.

Our results indicate limited biological differences between proton and 6 MV X-ray irradiation for HSPCs. However, the extrapolation of our in vitro data to the clinical level is not that obvious, as the in vivo bone marrow is still a complex environment for HSPCs and intra- individual differences in the radiosensitivity of HSPCs are present, due to the heterogeneity within this cell population and inter-individual differences in HPSCs radiosensitivity have also been shown^36,45^. Also, UCB is neonatal peripheral blood which contains HSPCs that are more immature and possess a higher proliferative potential, and exist in higher concentrations than HSPCs found in bone marrow^46^. Nevertheless, our results provide a first insight into the fundamental DNA damage induction and repair mechanisms of HSPCs exposed to different beam qualities. The next step towards clinical translation would be to establish a murine model to further confirm the limited biological differences between both irradiation exposures in vivo. Finally, prospective clinical trials are required to assess the *in human* acute toxicity risks and long-term risk of developing hematological malignancies after proton exposure for pediatric patients with CNS tumors.

The fact that proton therapy reduces the integral dose to the patient compared to X-ray radiotherapy will result in a lower exposure of surrounding normal tissue and reduced risk to develop side effects. For hematological toxicity, this was recently illustrated by Vennarini et al.^47^ showing no acute hematological toxicity during cranio-spinal proton therapy for pediatric embryonal tumors. Next to this, Liu et al.^48^ showed that proton cranio-spinal irradiation significantly decreased hematologic toxicity compared with those receiving photon therapy. Also, Ruggi et al.^12^ showed in pediatric patients treated for medulloblastoma that hematological toxicity was limited, even among high-risk patients who underwent hematopoietic stem-cell transplantation before proton therapy. For long-term risks, longer follow-up studies must be completed but current preliminary results on late effects and survival are certainly encouraging. Additionally, in the clinic other approaches to overcome the higher proton RBE in the distal part of SOBP include the use of different incident angles that selectively avoid proton beams stopping in or directly in front of the most radiosensitive healthy tissue^39^.

In conclusion, we showed similar DNA damage induction and repair between mid-SOBP protons and 6 MV X-rays in HSPCs while distal SOBP protons showed a higher number of MN in HSPCs compared to mid SOBP protons depending on the radiation dose. This research indicates possible changes of the in vivo biological response of proton therapy. Therefore, exposure to distal SOBP protons needs to be handled with extra caution during proton treatment planning. Further research is needed to fully understand the DNA damage response of HSPCs in more complex, in vivo animal models and more clinical studies are needed to collect data on acute and long-term outcomes after proton irradiation.

## Methods

### Collection and isolation of CD34^+^ cells

Informed consent for inclusion was received from all UCB donors, before participating in the study. This study was conducted in accordance with the Declaration of Helsinki, and the protocol and informed consent was approved by the Ethical Committee of Ghent University (2017/1621). In total, 46 UCB samples were retrieved from the Red Cross Blood bank. First, peripheral blood mononuclear cells were obtained by density gradient centrifugation (density: 1.077 g/mL; Lymphoprep™, Axis-Shield, Dundee, UK). Then, HSPCs were purified by using CD34^+^ immunomagnetic beads (human CD34^+^ Microbead kit, Miltenyi Biotec Inc., Bergisch Gladbach, Germany) according to the manufacturer’s guidelines. CD34+ purity and viability were checked as previously described by Engelbrecht et al.^35^. Flow cytometric analysis revealed an average CD34^+^ purity of 95.47% (SD: 3.62%) and a viability minimum was set at 95%. Isolated CD34^+^ HSPCs were cryopreserved in liquid nitrogen after resuspension in 90% fetal calf serum (FCS) (Gibco, Thermo Fisher Scientific Ltd., Waltham, MA, USA) and 10% dimethyl sulfoxide (Sigma-Aldrich, Saint Louis, MO, USA) and 24 h in a Mr. Frosty container, containing isopropanol, (Sigma-Aldrich) at − 80 °C.

### Culturing and irradiation of CD34^+^ cells

Cells were thawed by washing them two times in complete Iscove’s Modified Dulbecco’s Medium (cIMDM: IMDM (Gibco), 0.5% pen/strep (Gibco) and 10% FCS; 37 °C) (1500 rpm, 10 min, 37 °C). For each radiation exposure, the CD34^+^ cell suspensions were irradiated in 1.0 mL cryogenic vials (Clearline CryoGen ®, Cona, Italy), positioned perpendicular to the beam axis.

*Proton irradiation.* Two proton facilities were used to irradiate CD34^+^ cells in mid-SOBP (iThemba LABS, Cape Town, South-Africa & PARTREC accelerator facility, University Medical center Groningen, University of Groningen, Groningen, The Netherlands). To evaluate the difference between different proton SOBP positions, HSPCs were also irradiated at distal-SOBP at the PARTREC accelerator Facility.

*Photon irradiation.* 6 MV X-rays were used as the reference beam quality. For the cytokinesis-block micronucleus (CBMN) assay, a 6 MV LINAC at iThemba LABS was used. For the γ-H2AX foci assay, a 6 MV LINAC at Ghent University Hospital (Ghent, Belgium) was used.

Based on depth-dose curve measurements, correct positioning of cryovials, containing the CD34^+^ cell suspension, was assured by using specific in-house made sample holders and aligning them with lasers at both the proton and X-ray irradiation facilities. Physical parameters and additional information on both proton and photon beams can be found in Table 2.

**Table 2:** Physical parameters and phantom set-up information on the different beam set-ups used for proton irradiation and 6 MV X-ray irradiation.

Protons	Beam energy	Dose-rate (Gy/min)	Field size (mm)	Mid-SOBP depth (mm)	Dis-SOBP depth (mm)	SOBP width (mm)	50% range (mm)	Beam modulation
KVI-CART	190.0 MeV	1	100 o	200	221	41	221	Range shifter consisting of 8 × 2.5 mm Aluminium
IThemba LABS	199.5 MeV	3	100 o	85	–	31	100	Range-modulator wheel

X-Rays	Beam quality	Dose-rate (Gy/min)	Field Size (mm)	Sample depth in water (mm)	Linear Accelerator	Protocol for determination of absorbed dose		
IThemba LABS	Percentage Depth Dose 20/10: 0,7 10 × 10 field, SSD = 100 cm	2	100 × 100	50	Philips SL75-5	TRS-398		
University Hospital Ghent	Tissue Phantom Ratio 20,10: 0,69 10 × 10 field, SID = 100 cm	5	200 × 200	100	Elekta Synergy	NCS report 18 [REF]		

### The cytokinesis-block micronucleus assay

For this assay, the CD34^+^ cells were irradiated with 0.5, 1 or 2 Gy. A quantity of 200 000 CD34^+^ cells were cultured in 1 mL cIMDM and incubated (37 °C, 5% CO2) for 1 h before irradiation in a cryogenic vial. After irradiation, cells of one vial were seeded out in two wells of a 48-well suspension plate (Greiner Cellstar^®^, Sigma-Aldrich). The cells were stimulated into division and the CD34^+^ micro-culture CBMN assay was performed as recently described by Engelbrecht et al.^35^. The nuclear division index (NDI) provides a measure of the cell’s proliferative status. For each culture, 500 viable cells (N_total_) were scored to evaluate the number of mononucleate (N_1_), binucleate (N_2_), trinucleate (N_3_), and polynucleate (N_4_) cells. The formula used to calculate NDI: NDI = (N_1_ + 2N_2_ + 3N_3_ + 4N_4_)/N_total_. For this study, all samples’ nuclear division indexes were within the range of 1.2–2.2 (data not shown). Micronuclei (MN), representing mainly acentric chromosome fragments that are not incorporated in the main nuclei after cell division, were manually scored in 1000 binucleated (BN) cells for each dose point (500 BN cells/duplicate culture) using a fluorescence microscope (200 × magnification, Leica). Details can be found in addendum A: ‘Cytokinesis-block micronucleus Assay’.

### The γ-H2AX foci assay

For the γ-H2AX foci assay, 200 000 unstimulated CD34^+^ cells were incubated (37 °C, 5% CO2) for 1 h in a cryogenic vial containing 1 mL cIMDM. After irradiation, the content of each cryogenic vial was divided over two cryogenic vials which were placed back in the incubator (37 °C, 5% CO2). Cells were incubated for 30 min, 2 h and 24 h post-irradiation, to detect γ-H2AX foci, representing DNA DSB induction (30 min) and repair (2 h, 24 h). After incubation the cells were placed on ice for 15 min, followed by cytospinning 250 μL cell suspension, representing 50 000 cells, on duplicate slides for both cultures. Then, immunostaining for the γ-H2AX protein was performed. Details on the immunostaining can be found in addendum B: ‘γ-H2AX foci assay’.

After leaving the immunostained slides overnight at 4 °C, slides were scored automatically with the MetaCyte software module of the Metafer 4 scanning system (MetaSystems, Altlussheim, Germany) using a 63x-oil objective with the fluorescein isothiocyanate (FITC) filter (z-stage = 10). At least 500 CD34^+^ cells were scored over duplicate slides from duplicate cultures. Afterwards, cell selection was manually checked for artefacts. For each beam quality, the average number of radiation-induced γ-H2AX foci was obtained by subtracting the mean number of γ-H2AX foci present in the sham-irradiated sample from the mean number of γ- H2AX foci in the irradiated samples of the same donor. At both 30 min and 2 h post-irradiation, the 30 min sham-irradiated control was used. At 24 h post-irradiation, the 24 h sham-irradiated control was used.

### Biological enhancement ratio

Given the limited number of dose points in this study, a biological enhancement ratio was calculated instead of RBE. Biological enhancement ratio is the ratio of the radiation-induced biological effect (number of MN/1000BN cells or number of γ-H2AX foci) of proton irradiation relative to the reference irradiation (6 MV X-rays), for a specific radiation dose.

### Statistical analysis

Statistical analysis was performed using the Graphpad Prism 9.4.1. software (GraphPad Software Inc., San Diego, CA, USA). Shapiro–Wilk tests assessed normality of data and analysis of variance (ANOVA) with Tukey’s multiple comparisons test was used for the comparison of the number of radiation-induced MN/1000BN cells and the average number of radiation-induced γ-H2AX foci. Statistical significance was set at *p* < 0.05 (two-sided).

### Supplementary Information

Supplementary Information.

## Data Availability

All data generated and analyzed during this study are included in this published article (and its supplementary information files).

## Abbreviations

HSPCs: Hematopoietic stem and progenitor cells
DSB: Double-stranded break
CNS: Central nervous system
SOBP: Spread-out-Bragg peak
RBE: Relative biological effectiveness
LET: Linear energy transfer
UCB: Umbilical cord blood
FCS: Fetal calf serum
cIMDM: Complete Iscove’s modified Dulbecco’s medium
CBMN: Cytokinesis-block micronucleus
NDI: Nuclear division index
MN: Micronuclei
BN: Binucleated
FITC: Fluorescein isothiocyanate
DAPI: 4',6-Diamidino-2-fenylindool
ROS: Reactive oxygen species
