# Supplementary material for: Impact of proton therapy on the DNA damage induction and repair in hematopoietic stem and progenitor cells
Source: Sci Rep. 2023 Oct 9;13:16995. doi: 10.1038/s41598-023-42362-0 (PMC10562436; doi:10.1038/s41598-023-42362-0)
Supplement: Supplementary file 1 — Supplementary Information. [file 41598_2023_42362_MOESM1_ESM.pdf]

Supplementary Files (Addenda)

A. Cytokinesis-block micronucleus assay

Stem cell factor (SCF) (100 ng/mL, Peprotech, London, UK), Flt3-ligand (Flt3-L) (100 ng/mL, Peprotech) and thrombopoietin (TPO) (20 ng/mL, Peprotech) were added and cells were incubated as previously described [31]. At 23 h post-stimulation cytokinesis was blocked by adding 6 µg/mL cytochalasine B (Sigma-Aldrich*)* to every culture, to obtain BN cells. 70 h post-stimulation the cells were resuspended gently to reduce cellular clumping. Each well was rinsed with 0.5 mL phosphate buffered saline (PBS). After centrifugation (8 min, 300 g, Eppendorf, Hamburg, Germany) and supernatant removal, cells were exposed to a cold hypotonic shock (0.075 M KCl, 4°C) and were fixed overnight in a 3:1:4 (methanol/acetic acid/ ringer, 4°C) solution. The next day, cells were fixed twice in a 3:1 (methanol/acetic acid, 4°C) solution and left at 4°C overnight. Cell suspension was dropped on isopropanol-cleaned slides and allowed to air dry. After drying, fluorescent staining with acridine orange is performed (Stock solution: 0.1 g acridine orange powder in 100 ml aq.d.; Buffer: 1 acridine orange buffer tablet in 1 l aq.d.; Working solution: 0,6 ml acridine orange stock solution in 60 ml acridine orange buffer. 20 µL acridine orange buffer is applied before covering it with a coverslip and sealing it with rubber cement glue, to prevent the slides from drying out.

B. γ-H2AX foci assay

Cells were fixed in PBS containing 3% paraformaldehyde (PFA, VWR, Wayne, PA, USA) for 20 min, and were transferred to PBS (4°C) for overnight storage. Subsequently slides were washed in a bath of PBS for 5 min, followed by covering the cells with ice cold PBS-Triton X-100 (0.2%, Gibco) for 10 min. Thereafter, cells were washed 3x 10 min with PBS-BSA (1%). Immunocytochemistry was performed by a 1 h incubation with the γ-H2AX protein primary antibody (murine, monoclonal (Biolegend, San Diego, CA, USA), 1/500 dilution in PBS with 1% BSA). Subsequently, the cells were washed 3x 3 min with PBS-Tween20 (0.3%, VWR). The secondary antibody (Goat Anti Mouse, polyclonal, Dylight 488 (Thermo Fisher Scientific Ltd., Waltham, MA, USA), 1/1000 dilution in PBS with 1% BSA) was added to the cells and after 1 h incubation, the cells were washed another 3x 3 min with PBS-Tween20 (0.3%). After submersion in a PBS bath for 5 min, 40 µL fluoromount (Sigma-Aldrich) with 4',6-diamidino-2-phenylindole (DAPI) (Sigma-Aldrich, 200 ng/mL) was added and a clean coverslip was applied.
